# Consequences of climate-induced vegetation changes exceed those of human disturbance for wild impala in the Serengeti ecosystem

**DOI:** 10.1093/conphys/coz117

**Published:** 2020-01-21

**Authors:** L Hunninck, R May, C R Jackson, R Palme, E Røskaft, M J Sheriff

**Affiliations:** 1 Department of Biology, Norwegian University of Science and Technology, Høgskoleringen 5, 7034 Trondheim, Norway; 2 Norwegian Institute for Nature Research, Høgskoleringen 9, 7034 Trondheim, Norway; 3 Department of Biomedical Sciences, University of Veterinary Medicine, Veterinärplatz 1, 1210 Vienna, Austria; 4 Biology Department, University of Massachusetts, 285 Old Westport Road, Dartmouth, MA 02747, USA

**Keywords:** conservation, cortisol, forage quality, NDVI, protected areas, stress, ungulate

## Abstract

In East Africa, climate change is predicted to reduce vegetation quality, and pervasive human disturbance has already resulted in significant declines in biodiversity. We studied the combined effects of reduced forage quality and human disturbance on faecal glucocorticoid metabolite (FGM) concentrations. We predicted that decreasing nutritional quality and increasing human disturbance would have an additive positive effect on FGM levels in wild impala (*Aepyceros melampus*). Employing a space-for-time approach, we used normalized difference vegetation index (NDVI) as a measure of forage quality, combined with spatially explicit proxies of human disturbance across areas of different protection management strategies in the Serengeti ecosystem. We collected 639 faecal samples, spread over 4 years, including both wet and dry seasons. Impala FGM levels increased significantly with declining NDVI and, to a lesser extent, with increasing proxies for human disturbance. However, we found no interaction between the two, such that impala had elevated FGM levels with low NDVI and low FGM levels with high NDVI regardless of human disturbance levels. This implies that impala will have high FGM levels if forage quality is poor, even with significant protection and reduced human disturbance. Understanding how animals respond to and cope with changes in forage quality and human land use across different protected areas is important for conservationists and managers to better protect species at risk and predict population viability.

## Teaser

Climate change and human disturbances are major threats to biodiversity. We studied the interactive effects of reduced forage availability together with human disturbance on glucocorticoid (GC) levels in wild impala. Our results show that impala had elevated GC levels when forage quality was low, even with significant protection and reduced human disturbance.

## Introduction

Global biodiversity is in decline, caused primarily by anthropogenically induced changes in climate and land use (Pimm *et al.*, 2014; Johnson *et al.*, 2017). Anthropogenic disturbances now significantly impact nearly every habitat on Earth, and human-induced rapid environmental changes are forcing many species to either adapt at an unprecedented pace or perish (Sievers *et al.*, 2018). Organisms may adapt to these disturbances through behavioural, physiological and/or morphological mechanisms (Sih *et al.*, 2011). While certain species thrive in these new, human-altered environments, most species face population declines, with some researchers predicting that a large proportion of the Earth’s biodiversity will be extinct by 2100 (Stork, 2010; IPBES, 2018). In East Africa, climate change is severely altering weather patterns and could significantly reduce forage quality (Boko *et al.*, 2007; Niang *et al.*, 2014). Furthermore, exceedingly pervasive human land use and land cover change, mainly due to agricultural expansion, is considerably changing and reducing the region’s natural habitat (Willcock *et al.*, 2016). The reduction in forage quality through the combined effect of climate-induced and human land cover change poses a significant threat to the region’s biodiversity (Midgley and Bond, 2015; Segan *et al.*, 2016). In the Serengeti-Mara ecosystem, wildlife populations have declined dramatically, especially in areas with high human disturbance (Ogutu *et al.*, 2009; Veldhuis *et al.*, 2019). Although protection measures have been implemented, including the creation of protected areas such as Serengeti National Park (SNP), understanding how animals respond to and cope with declines in vegetation quality and increased human land use across areas with different protection strategies is important for conservationists and managers to better protect species at risk and predict population viability.

Ungulate populations are to a large extent regulated by forage quality (Hopcraft *et al.*, 2010). In the east African savanna, grass growth is mainly regulated by soil fertility and rainfall (Bartzke *et al.*, 2018) and is characterized by strong seasonality. Grasses in these savanna ecosystems periodically dry and become less nutritious for herbivores (Codron *et al.*, 2007). Animals can adapt to decreased forage quality by either migrating to better grazing patches or adjusting their diet (Hopcraft *et al.*, 2010). For example, the Serengeti ecosystem is able to sustain more than one million blue wildebeest (*Connochaetes taurinus*) because most migrate between the northern and southern part of the ecosystem, drawn to fresh pastures which appear after the first rains (McNaughton and Banyikwa, 1995; Hopcraft *et al.*, 2013). Impala (*Aepyceros melampus*), on the other hand, are sedentary and thus must forage on a mixed diet, preferring nutritious grasses but needing to include more browse in their diet as grasses dry out (Jarman and Jarman, 1973; Dunham, 1982). The seasonal fluctuations in rainfall in eastern Africa, and thus forage quality, are predicted to become more extreme with increasingly severe climate change (Dore, 2005; Sinclair *et al.*, 2007; Midgley and Bond, 2015), potentially resulting in prolonged drought periods (Dai, 2011; Kotir, 2011) and significant reductions in nutritious grasses across savanna habitat (Stevens *et al.*, 2016).

As a proxy of spatiotemporal variability in forage quality, we used the normalized difference vegetation index (NDVI; NASA MODIS; Didan, 2015). NDVI is a measure of primary productivity or greenness of vegetation cover calculated from the amount of red and near-infrared light reflected from the Earth’s surface (Pettorelli *et al.*, 2005). NDVI is a commonly used metric for changes in primary production, though care should be taken with the interpretation of the results (Pettorelli *et al.*, 2011). For example, changes in plant species composition or habitat structure can significantly affect the interpretation of NDVI values in space. As such, it is advised to only compare changes within the same habitat, and not between different ecosystems (Pettorelli *et al.*, 2005). Here, we employ NDVI within areas specific to our study species in the African savanna ecosystem, mainly consisting of a particular grassland and woodland mosaic.

In African savanna ecosystems, ungulates also face increasing pressure from anthropogenic disturbances. Some human activities, such as infrastructure and tourism, invoke a multitude of behavioural responses which can sometimes be so pervasive they impact population viability (Frid and Dill, 2002; Szott *et al.*, 2019). For example, African elephants (*Loxodonta africana*) and impala adjust their diurnal activity or movement patterns to limit exposure to these human activities (Wronski *et al.*, 2015; Gaynor *et al.*, 2018). Additionally, several studies have shown that animals in strictly protected areas such as national parks have lower glucocorticoid (GC) levels than their conspecifics in less protected areas, implying that GC levels might be a good indicator for protection level of an area (Ahlering *et al.*, 2011; Spercoski *et al.*, 2012; Hunninck *et al.*, 2017). Human-induced changes in land use and cover have also contributed considerably to the degradation of natural grasslands in east Africa, particularly in areas with high agricultural and pastoral activities (Laurance *et al.*, 2014), resulting in a decline in overall vegetation productivity (Landmann and Dubovyk, 2014). Growing livestock numbers increase resource competition with wild ungulate populations and result in habitat modifications (Prins, 2000; Young *et al*., 2005; but see Schuette *et al*., 2016). Together, reduced forage quality combined with changes in human land use are predicted to pose the biggest threat to wildlife in eastern Africa, and a better understanding of their impact on animal populations is needed (Vié *et al.*, 2009; Niang *et al.*, 2014).

The physiological stress response is an essential part of vertebrates’ ability to cope with and respond to challenges in their environment (Boonstra, 2013). One part of this response is through the activation of the hypothalamic–pituitary–adrenal (HPA) axis and subsequent secretion of GCs into the blood stream (Romero, 2004). Although GCs affect a range of bodily functions, their primary role is energy mobilization (Strack *et al.*, 1995). Upregulating the secretion of GCs allows animals to mobilize the energy needed—even at the cost of tissue mass—to facilitate the required physiological and behavioural responses needed for organisms to mitigate a stressor (Romero and Wingfield, 2015). These temporary changes allow organisms to better deal with adverse situations (i.e. stressors; MacDougall-Shackleton *et al.*, 2019), such as increased predation pressure or food deprivation (Sheriff *et al.*, 2011a; Dantzer *et al.*, 2014), by, among other, increasing energy availability for muscles, and suppress anabolic processes non-essential for short-term survival such as growth, reproduction and digestion. The adaptive value of this energy mobilization under threat helps by both diverting energy where it is needed while enhancing recovery and preparation for a repeated stressor (Sapolsky *et al.*, 2000). However, if the stressor is frequently recurring or constant over a longer time span (i.e. chronic stressor), this adaptive stress response can result in adverse effects for the organism, such as suppressed growth, lower immune function, increased energy expenditure, and potentially reduced reproduction and survival (Busch and Hayward, 2009; Romero and Wingfield, 2015). Thus, the measurement of GCs may provide a robust assessment of animals’ overall health, their ability to cope with changes within their environment, and the potential fitness consequences of their responses (Sheriff *et al.*, 2011a; Dantzer *et al.*, 2014).

In this study, we tested the hypothesis that decreased forage quality and increased anthropogenic land use would significantly increase GC levels in wild impala within the Serengeti ecosystem. Impala are a common herbivore in this system and, due to their small home ranges, high local abundance and non-migratory behaviour, are an ideal model species to study the effect of spatially explicit disturbances on an animal’s adrenocortical activity. To test our hypothesis, we used NDVI and spatially explicit proxies of human disturbance across areas of different protection management strategies, including SNP (see Methods). This allowed us to study the interactive effects between forage quality and human disturbances on faecal glucocorticoid metabolite (FGM) levels of a wild ungulate. Specifically, we predicted that impala would have significantly higher FGM levels (i) in areas with reduced forage quality as measured by lower NDVI scores, (ii) in areas with greater human disturbance, measured as settlement density, and especially (iii) in areas with reduced forage quality and high human disturbance. We also predicted that the protection status of an area would influence impala FGM levels, such that (i) impala in adjacent areas but near SNP would have lower FGM levels that those further away and (ii) impala in areas with greater protection status would have lower FGM levels.

## Methods

### Study area and species

**Figure 1:**
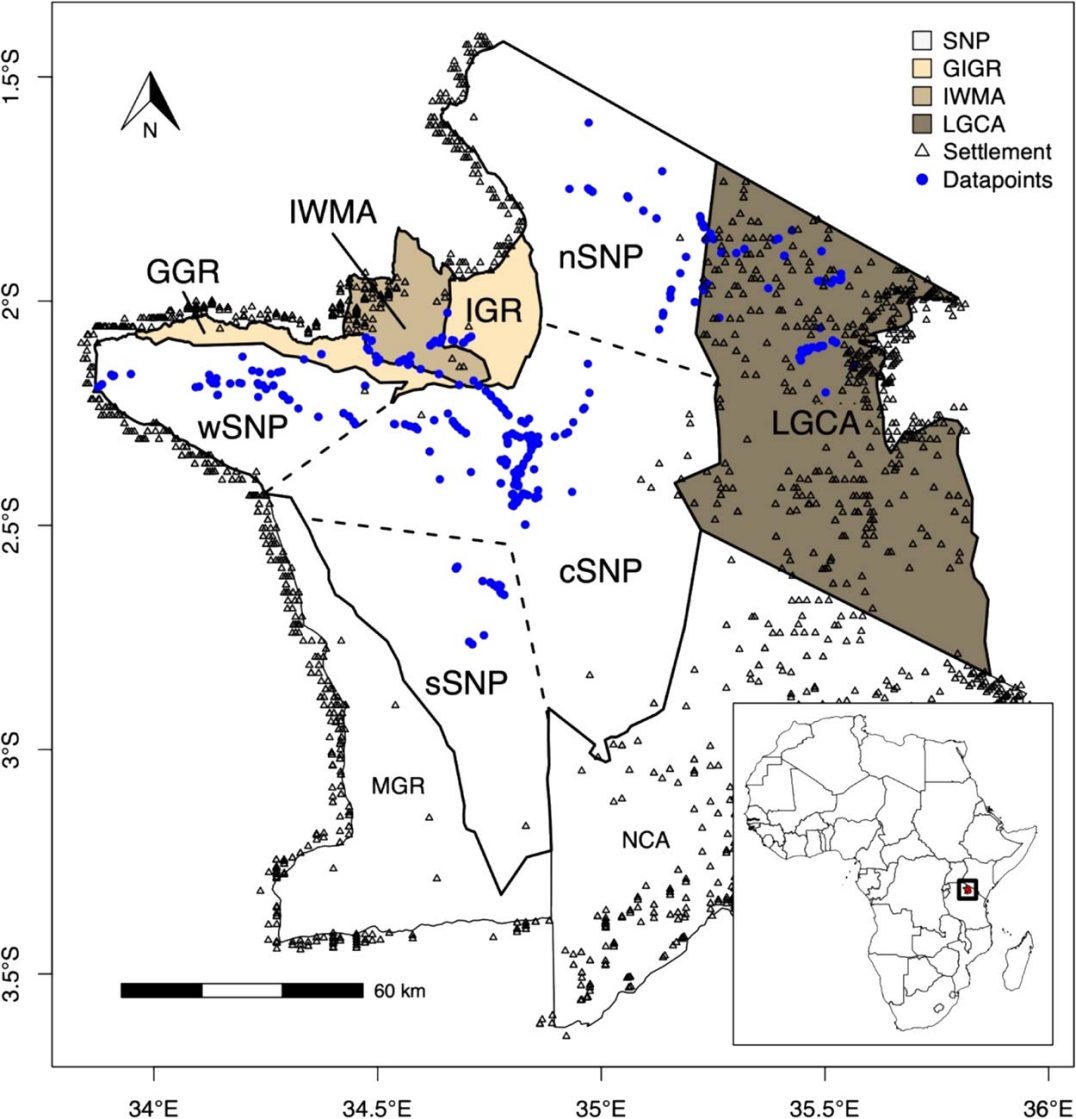
**The Serengeti ecosystem.** Map of the Serengeti ecosystem, consisting of seven areas with different management strategies and different human land uses. Serengeti National Park (SNP) is subdivided in four areas by dashed lines (see Methods). Areas with darker fills are areas with higher predicted intensities of human disturbance. Locations where samples were collected are in solid (blue) circles, while settlement locations are represented as open triangles

The Serengeti ecosystem (±27 000 km^2^) experiences high geographic variability in rainfall, from around 450 mm in the southeast to > 1400 mm in the north; rainfall comes in two separate wet seasons (March–May and November–December). The ecosystem consists of seven areas with different management strategies and human land use; our study was limited to five of these areas (Fig. 1); SNP, Grumeti and Ikorongo Game Reserves (GIGR), Ikona Wildlife Management Area (IWMA) and Loliondo Game Controlled Area (LGCA). Of these, SNP has the highest levels of protection and extractive activities such as hunting and livestock grazing are strictly prohibited. Tourism, traffic and illegal activities such as poaching (i.e. illegal bushmeat hunting) are considered the main human disturbances in the park, as settlements are not allowed (Nyahongo *et al.*, 2005). We distinguished four subareas within SNP because of their differences in intensity of human activities: central (cSNP; high tourism, low poaching), west (wSNP; high poaching, medium tourism), north (nSNP; low tourism, low poaching) and south (sSNP; medium tourism, medium poaching) (Loibooki *et al.*, 2002; Lindsey *et al.*, 2013). GIGR is our medium protected area; it allows licensed hunting and tourism, but no settlements or agropastoralism. IWMA and LGCA have the lowest protection; they allow settlements, licensed hunting in designated hunting blocks and agropastoralism. The cumulative effect of different human disturbances is particularly difficult to estimate and compare; however, we expect LGCA to have the highest level of human disturbance, followed by IWMA, GIGR and lastly the areas inside SNP. SNP has comparatively low human disturbance (although the number of tourists is increasing), and this was expected to be similar in cSNP, sSNP and nSNP but higher in wSNP, due to potentially higher poaching levels.

Impala are a medium-sized antelope species common in eastern and southern African savanna ecosystems (IUCN SSC Antelope Specialist Group, 2016). Impala are non-migratory herbivores with small home ranges typically between 5 and 10 km^2^, increasing only slightly in the dry season (Averbeck, 2001). They are often found on the edge of open savanna as their preferred habitat is open woodland (Ford *et al.*, 2014). Their habitat requirements result in impala having a clumped and irregular distribution, but locally abundant (Averbeck, 2001). In East Africa, impala males are territorial year round (Oliver, 2005) and male–male aggression likely elicits a stress response (e.g. Corlatti, 2018).

### Collection and analysis of faecal samples

To assess GC levels in impala, we measured FGMs. FGMs reflect the biologically active free plasma GCs (Sheriff *et al.*, 2010), and sample collection is non-invasive (Sheriff *et al.*, 2011a; Madliger *et al.*, 2018). FGMs are an integrative measure of plasma GCs (±2 h in impala), representing an average value rather than a point value of GC levels (Palme, 2019).

We collected 639 samples from individual adult impala (499 females, 140 male) across five collection periods, spanning 4 years (2012, 2016, 2017 and 2018) in both wet and dry seasons (Supplementary Table S1). When a suitable individual was seen defecating, a picture was taken and the distance to the individual was recorded with a range finder. This method allowed us to easily identify the specific sample (Lunde *et al.*, 2016). The sample was not collected when two or more samples were close to each other (within 1 m). For each faecal sample that was collected, we recorded the sex of the individual from whom the sample came (adult males have horns), and the size and type (family [one territorial male, females and juveniles], bachelor [only adult and subadult males], or mixed [when family herds mixed with bachelor herds]) of social group. We also took a GPS location of the collection site and habitat and noted the time of day. Habitat was categorized into four different types; grassland (grass dominated with < 2% tree canopy), savanna (grassland with < 20% tree cover), woodland (>20% tree cover, defined as trees > 6 m with canopy cover 20% or higher) and bushland (dense woody vegetation < 6 m in height with > 20% bush canopy). We could sample individuals from multiple groups from a single location in a single day; however, we did not return to the same location within a collection period to avoid potential pseudo-replication, i.e. resampling the same individual. Samples were collected within 60 min of defecation (mean ± SD = 28 ± 14 min) and immediately placed on ice and, within 12 h of defecation, stored at −20°C until further analysis.

### Analysis of FGMs

FGMs were analyzed using a group specific enzyme immunoassay (EIA) according to Palme (2005) and Touma and Palme (2005). Briefly, faecal samples were defrosted at room temperature for 30 min and homogenized by hand for 5 min. A portion of 0.52 ± 0.023 g (mean ± SD) of homogenized faeces were mixed with 5 ml of 80% methanol and vortexed for 1 min. Samples were then centrifuged for 20 min at 2500 *g*, and 0.5 ml of supernatant was removed. Samples were then placed in a fume hood for up to 48 h to allow methanol to evaporate. Samples were then sealed and stored at −20°C until shipment and analysis at the University of Veterinary Medicine, Vienna, Austria. FGMs were measured with an 11-oxoetiocholanolone EIA, first described by Möstl *et al*. (2002) which measures metabolites with a 5β-3α-ol-11-one structure. This EIA has been specifically validated for impala (Chizzola *et al.*, 2018). Intra-assay variations of high- and low-value quality controls were 5.27 and 5.76%, respectively, and inter-assay coefficients of variation of high- and low-value quality controls were 10.39 and 12.15%, respectively.

### Collection of remote sensed NDVI data

The data were retrieved from the online Application for Extracting and Exploring Analysis Ready Samples (AppEEARS), courtesy of NASA (https://lpdaacsvc.cr.usgs.gov/appeears/). Using the pixel reliability dataset that accompanies the NDVI data, pixels containing clouds were filtered out. NDVI data was adjusted to account for empty data points and outliers using a Savitzky–Golay smoothing filter.

NDVI measurements should only be compared within the same habitat, and not between different ecosystems (Pettorelli *et al.*, 2005). However, impala are most often found in very similar habitat, regardless of area, preferring semi-open to bushy savanna and rarely venturing far from the cover of woody vegetation (Jarman and Jarman, 1973; Ford *et al.*, 2014). To assess habitat differences in sample locations, we used remotely sensed data on woody cover (MOD44B MODIS/Terra; Dimiceli *et al*., 2015). As expected, woody cover percentage at sample locations was low (mean ± SD = 5.6% ± 3.2; *N* = 693) and adjusting NDVI for % woody cover did not affect model estimates (see Supplementary Information S1). Therefore, although NDVI measures greenness of vegetation of both woody and non-woody plants, in this data set, variation in NDVI is mostly due to variation in grassy vegetation. Thus, here, NDVI correlates positively with the abundance of grassy vegetation, and since grassy vegetation is considerably more palatable than browse and therefore preferred by impala (Jarman and Jarman, 1973; Codron *et al.*, 2007), NDVI represents an unbiased proxy for forage quality for impala (Pettorelli *et al.*, 2011).

For each of the faecal samples collected, we extracted the closest NDVI value in space (250 m MODIS pixel resolution) and time (8-day interval). Thus, we acquired an NDVI score specific to our faecal sample with regards to location and time of collection. Considering the limited movement of impala, impala equipped with a GPS collar moved on average 262 m in 3 h (SD = 247, *N* = 212 000; *unpublished data*) away from their initial location; this NDVI score provides a reasonable representation of the environment utilized by the sampled impala over the past week (well within the integrated hormone levels experienced by each individual).

### Collection of remote sensed rainfall data

To estimate the potential effect of rainfall on FGM concentrations in impala, we collected data from the CHIRPS dataset (Funk *et al*., 2015). This dataset has a temporal resolution of 1 day and a spatial resolution of 5 km resolution; data was downloaded from ClimateSERV (https://climateserv.servirglobal.net/). We calculated the cumulative rainfall over a 7-day period prior to sample collection for each sample (t0 to t-7, with t0 = time of sample collection), specific to its location. The rainfall data was zero-inflated as many samples were collected in the dry season and was therefore converted in a categorical variable with three levels: ‘No rainfall’ (Rainfall = 0; *N* = 287), ‘Low rainfall’ (0 < Rainfall ≤ 12; *N* = 151) and ‘High rainfall’ (Rainfall > 12; *N* = 201). The third quantile of the rainfall data was 12 mm and was therefore chosen as a threshold.

### Human disturbance

The Tanzanian Wildlife Institute provided data on settlement locations, which included bomas (i.e. used by pastoralists to protect their livestock), thatch roof huts and iron sheet huts/houses in and around most of the Serengeti ecosystem (Fig. 1; TAWIRI, 2016). Some settlements are located within the national park which is not allowed but does happen (Fig. 1). However, since a Kernel density estimation (KDE) was applied to the data, isolated points had little effect on the overall settlement density score. The specific settlement density score for each faecal sample was extracted and, after scaling the data (mean ± SD ≈ 0 ± 1), used for analyses. Distances to SNP boundary were calculated as the shortest Euclidean distance from the GPS location of each data point to the nearest park boundary (mean ± SD = 15.0 ± 9.7 km). Seven areas, four in SNP (i.e. cSNP, wSNP, nSNP and sSNP), GIGR, IWMA and LGCA, with different human activities and disturbances were recognized in this study (Fig. 1; shapefiles available on https://www.protectedplanet.net/). Accurate data for relevant human disturbance proxies are particularly difficult to come by, especially in high temporal and spatial resolution. Here, our human disturbance proxies do not have temporal variation; however, the inter-annual variation in these stressors is unlikely to have changed dramatically other than having become more intense. Therefore, we believe that these proxies still present a highly relevant insight in spatially explicit patterns of human disturbance on FGM levels.

### Statistical analyses

We constructed multiple linear mixed models using the *lmer* function of the *lme4* package v.1.1–17 in R (Bates *et al.*, 2015). The response variable, FGM, was log-transformed to obtain normal distribution of model residuals. The following fixed predictors were all included in the basic model: (i) NDVI as measure for forage quality, and (ii) settlement density, distance to SNP border and land use area as measure for human disturbance. Lastly, time of day was included in the model as a fixed predictor because research has shown that it is important to either account for time-of-day in the study design (i.e. collect samples at similar times of the day) or control for this confounder by including it as a predictor of FGMs (Palme, 2019; *see*Supplementary Information S2). We used a quadratic function to model time of day and distance to SNP border; a decision supported by model selection criteria (∆AICc < 2; Akaike information criterion adjusted for small sample sizes; Burnham and Anderson, 2002). Random effects included group number nested within sampling location, and collection period as a crossed random effect. This way, we accounted for differences between groups, spatial location and collection period (Table 1).

**Table 1:** **Model estimates from the final mixed effects model explaining the variation in faecal glucocorticoid metabolite concentrations in impala.** See text for further details

**Fixed effects**	Estimate	SE	df	*t* value	*P* value	
*(Intercept)*	7.27	0.32	19.37	22.89	<0.001	^***^
NDVI	−3.08	0.63	155.34	−4.85	<0.001	^***^
Settlement density	0.33	0.10	37.91	3.26	0.002	^**^
Distance to SNP (lin.)	−1.31	3.19	17.66	−0.41	0.686	
Distance to SNP (qua.)	−5.94	1.75	18.15	−3.40	0.003	^**^
Land use area						
wSNP	−0.21	0.21	25.04	−0.99	0.332	
nSNP	−0.60	0.21	18.56	−2.83	0.011	^*^
sSNP	0.48	0.27	12.87	1.79	0.097	.
GIGR	−0.59	0.28	25.84	−2.14	0.042	^*^
IWMA	−0.15	0.29	20.89	−0.52	0.607	
LGCA	−0.65	0.28	37.43	−2.37	0.023	^*^
Time-of-day (lin.)	−1.11	0.98	304.71	−1.13	0.260	
Time-of-day (qua.)	2.10	0.97	299.35	2.16	0.032	^*^
Rainfall						
Low	−0.06	0.14	254.26	−0.44	0.663	
High	−0.25	0.11	304.35	−2.38	0.018	^*^
**Random effects**	Variance	SD				
Group ID: location	0.24	0.49				
Location	0.03	0.19				
Sampling period	0.16	0.40				
Residual	0.28	0.53				

Significance codes: P < 0.001 ^***^; 0.001–0.01 ^**^; 0.01–0.05 ^*^; 0.05–0.1.

Several potential confounding factors were identified (but see Supplementary Information S2): sex, group size and type, the interaction between sex and group type, distance to the nearest road, habitat and rainfall. By comparing AICc values, we determined which of these confounding factors, when added to the basic model, significantly improved the variation explained by the model (∆AICc < 2); only rainfall significantly improved the model and was therefore included in the final model. Residuals were visually checked for normality and heteroskedasticity, and a multicollinearity was assessed with a generalized variation inflation factor (GVIF) analysis, which is a measure of the harm done by collinearity among predictors (Fox and Weisberg, 2011). No heteroskedasticity was found, and residuals were normally distributed; GVIF values corrected for the degrees of freedom (GVIF^[1/(2^*^df)]) were all lower than 1.8 (*vif* function of the *car* package v.3.0-0 in R (Fox and Weisberg, 2011)), which is well below the conservative threshold of 3 (Zuur *et al.*, 2010). The correlation matrix of fixed predictors is presented in Supplementary Table S2.

To test for the interactive effect of forage quality and human disturbance on FGM levels in impala, we added an interaction term to the final model between NDVI and settlement density (now called ‘interaction model’). We compared AICc values of both models to determine whether the addition of the interaction would improve the fit of the model.

All statistical analyses were performed in the statistical program *R*, v.3.5.0 (RCoreTeam, 2018), using RStudio v.1.1.453 (RStudio, 2016). Back-transformed model estimates are shown in all figures; plots illustrate adjusted response values, which show the relationship between the fitted response and a single predictor, with the other predictors averaged out. The *Y*-axis in the figures are truncated at 1000 ng/g to aid the presentation of results.

## Results

Our final model explained a large proportion of the variation in impala FGM concentrations (*conditional R^2^* = 72.0%; Nakagawa & Schielzeth 2013); the main predictors in the model (i.e. fixed effects: NDVI, Settlement density, Distance to SNP, Land use area, Rainfall and Time-of-day) explained (*marginal R^2^*) 28.3% of FGM concentration variation.

**Figure 2:**
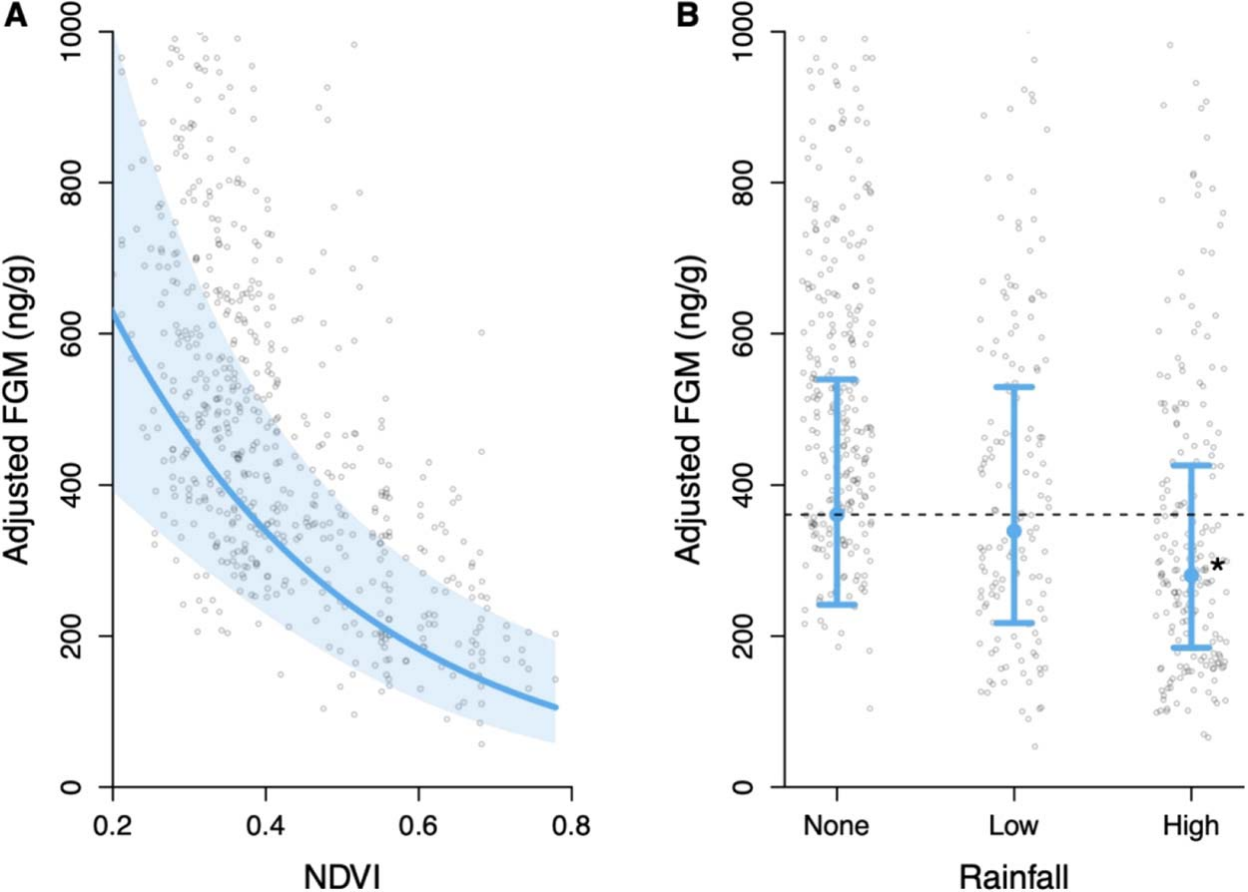
**Changes in impala FGM concentrations due to environmental factors.** The effect (blue line) of (**A**) the normalized difference vegetation index (NDVI), and (**B**) rainfall on impala faecal glucocorticoid metabolite (FGM) concentrations. Adjusted response values are represented as points; 95% confidence interval is the shaded blue area. On panel B, star denotes significant difference from no rainfall category (dashed line; *P* < 0.05).

We found that impala had significantly higher FGM levels in areas with lower NDVI scores (Table 1), such that mean FGM levels increased from 106 ng/g (95% confidence interval (CI) = 58–194 ng/g) at the highest NDVI values to 632 ng/g (CI = 394–1015 ng/g) at the lowest NDVI values (Fig. 2A). Rainfall (range: 0–27.5 ml) had a significant negative effect (Table 1) and mean FGM were highest (361 ng/g, CI = 241–539 ng/g) with no rainfall, and lowest (280 ng/g, CI = 185–426 ng/g) with relatively high rainfall (mean ± SE = 17 ± 0.37 mL; Fig. 2B).

**Figure 3:**
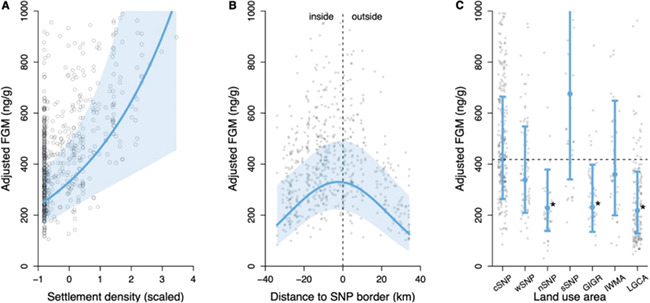
**Changes in impala FGM concentrations due to environmental factors.** The effect (blue line) of (**A**) Settlement density (kernel density estimate; scaled), (**B**) shortest Euclidean distance to nearest boundary of Serengeti National Park (SNP; dashed line) in kilometre and (**C**) land use area on impala faecal glucocorticoid metabolite (FGM) concentration. Adjusted response values are represented as points; 95% confidence interval is the shaded blue area or the error bars. On panel C, stars denote significant difference from cSNP (dashed line; *P* < 0.05).

FGM levels were significantly higher in areas with greater settlement density (Table 1), such that mean FGM levels increased from 252 ng/g (CI = 167–380 ng/g) at lowest settlement density to 1050 ng/g (CI = 456–2413 ng/g) at highest settlement density (Fig. 3A). Furthermore, we found that impala had significantly higher hormone levels at the border of the SNP (330 ng/g, CI = 222–491 ng/g; Table 1), while hormone levels decreased as distance to border increased whether inside or outside of the park (Fig. 3B). Management strategies across the region did not influence impala FGM levels as predicted (Fig. 3C; Table 1). Based on the management strategies, impala FGM concentrations in cSNP, sSNP and nSNP were expected to be similar, but lower than wSNP. Higher FGM values were expected in GIGR followed by IWMA and lastly LGCA. However, impala in sSNP tended to have the highest FGM levels (676 ng/g, CI = 340–1342 ng/g), followed equally (i.e. no significant difference these areas) by impala living in cSNP, wSNP and IWMA (m_cSNP_ = 418 ng/g, CI_cSNP_ = 263–664 ng/g). Impala in LGCA, GIGR and nSNP had the lowest FGM levels (m_LGCA_ = 218 ng/g, CI_LGCA_ = 128–371 ng/g; Table 1).

Impala mean FGM levels were significantly higher at dawn (6 am; 572 ng/g, CI = 333–983 ng/g) and dusk (6 pm; 413 ng/g, CI = 257–665 ng/g) and lowest at noon (1 pm; 323 ng/g, CI = 218–479 ng/g; Table 1). However, we accounted for this variation in our analysis and thus, these findings do not confound our results. Additionally, although FGM levels were significantly higher in territorial males compared to bachelors, adding this as a separate variable in the basic model did not improve the model fit and was therefore excluded.

**Figure 4:**
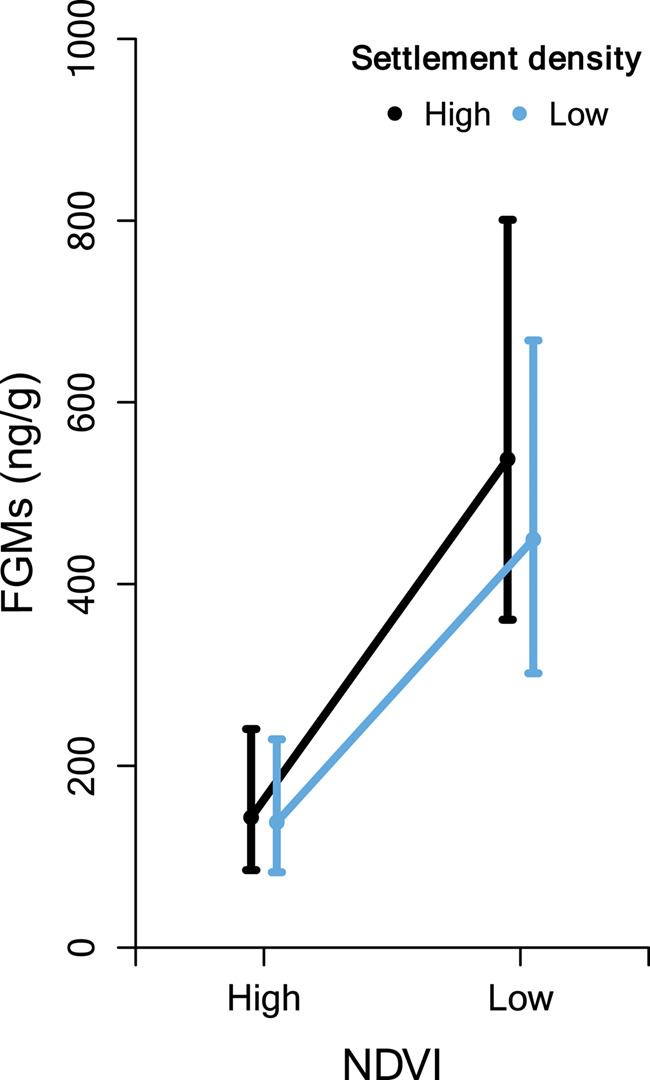
**Interaction between NDVI and settlement density on impala FGM concentrations**. The effect of high and low normalized difference vegetation index (NDVI) values on impala faecal glucocorticoid metabolite (FGM) concentrations when in high (black line) and low (light coloured line) settlement density (SD). High values are those higher than third quantile, and low values are those lower than first quantile. Includes 176 data points: 45 high SD and NDVI; 34 high SD, low NDVI; 40 low SD and high NDVI; 53 low SD and NDVI. Error bars show standard error of the estimate.

Importantly, since the interaction model had a ∆AICc value of 1.03 compared to the final model and adhering to the principle of parsimony, this means that the addition of the interaction term did not significantly improve the amount of variation in FGM explained by the model. We therefore conclude that there was no support for an interaction between NDVI and settlement density in our data (Fig. 4). The most influential predictor was NDVI, regardless of human disturbance levels; NDVI alone explained as much as 20% of the variation in impala FGM concentrations.

## Discussion

We tested the hypothesis that forage quality and anthropogenic land use would significantly affect FGM levels in wild impala. As predicted, impala experiencing lower forage quality had elevated FGM levels. Impala FGM concentrations increased with heightened levels of human disturbance, but levels differed unexpectedly in areas with different management regimes. There was no interaction between NDVI and settlement density, and our results show that NDVI was the most important factor predicting FGM levels in impala, regardless of human disturbance.

### Forage quality

We found that impala FGM levels significantly increased with decreasing NDVI (Figs. 2A and 4). The Serengeti ecosystem is a semi-arid savanna habitat (Sinclair *et al.*, 2008), and the nutrient-rich grassy vegetation recedes drastically during the dry season, forcing impala to include more browse in their diet. This corroborates previous findings that GC concentrations correlate negatively with food abundance (Busch and Hayward, 2009). To our knowledge, NDVI has only twice been used as a proxy for forage quality in relation to FGMs in wild ungulates. Stabach *et al.* (2015) found a strong negative relation between the change in NDVI over 2 weeks and FGMs in blue wildebeest, indicating that nutrient poor dry or senescent grass may lead to higher FGM concentrations in wildebeest. FGM levels of Asian elephants (*Elephas maximus*) were found to negatively correlate with NDVI values (Pokharel *et al.*, 2018). Similarly, even when controlling for the effect of predation pressure, song sparrows (*Melospiza melodia*) were found to have significantly higher GC levels when experiencing low food abundance (Clinchy *et al.*, 2004). Thus, we expect that it is a shift to a less nutrient-rich diet when NDVI is low that results in greater FGM levels for impala.

We also found that impala FGM levels were significantly higher when there had been no rainfall in the past week, compared to when there was relatively high rainfall (Fig. 2B). Droughts are associated with reduced forage quality for impala, as grassy vegetation recedes drastically during extended period of no rainfall. That impala are sensitive to climatic conditions, having the greatest FGM levels in areas with poor vegetation and drought like conditions, was expected. In red deer (*Cervus elaphus*), variation in FGMs was better explained when including stochastic weather events, such as flash floods, indicating that such weather events might be relevant environmental stressors (Corlatti *et al.*, 2011).

Climate change is predicted to have severe effects in eastern Africa, with higher temperatures and increased variability in rainfall potentially leading to increased number of inclement weather events and seasonal declines in abundance of nutrient-rich grasses (Niang *et al.*, 2014). We found that impala experienced elevated FGM levels when forage quality was low, and when rainfall was absent, and therefore FGM levels are likely to further increase in the future. Additionally, since forage quality is an important predictor of reproductive success (Parker *et al.*, 2009), a decline in green, nutrient-rich vegetation through both climate and human land use change is likely to impact population persistence of impala and other herbivores, especially exclusive grazers who cannot shift their diet to include more browse (Parker *et al.*, 2009).

### Human disturbance

Impala FGM concentrations increased with increasing settlement density (Fig. 3A). Increasing human density is associated with both direct human–wildlife conflicts and indirect human effects such as increased competition with livestock. For example, impala may adjust their daily activity in areas with higher human disturbance, reducing daytime activity, increasing afternoon activity and omitting their midday rest (Wronski *et al.*, 2015). Time spent vigilant, which is considered a costly behaviour, increases in impala and other ungulates in relation to human disturbances (Caro, 2005; Setsaas *et al.*, 2018). Similarly, GC concentrations can increase in ungulates due to human-related disturbances such as infrastructure and traffic (Creel *et al.*, 2002; Formenti *et al.*, 2018), and livestock and human presence (Stabach *et al.*, 2015). Lunde *et al*. (2016) found that impala in the Serengeti ecosystem had elevated FGM concentrations in relation to increased road type and traffic.

Furthermore, in areas with higher livestock densities, impala and livestock are likely competing for limited resources, especially during the dry season, adjusting their behaviour and thus increasing the energetic cost to obtain nutritious forage (Odadi *et al.*, 2011). Cattle in particular have been shown to suppress wildlife populations (Riginos *et al.*, 2012). We suggest that this increased habitat and forage competition with livestock, together with increased interactions with humans, results in an increased energy expenditure to obtain sufficient resources, and thus increased FGM concentrations in impala.

Impala FGM concentrations significantly increased with increasing proximity to the SNP border, regardless of whether impala were inside or outside of the park (Fig. 3B). We expected FGM levels to be lowest inside the park and increase with increasing distance from the park boundary. African elephants exhibited elevated FGM levels outside of protected areas, compared to inside (Tingvold *et al.*, 2013; Hunninck *et al.*, 2017), and lions (*Panthera leo*) had lower FGM concentrations when residing inside a conservation area, compared to those in a buffer zone with human settlements (Creel *et al.*, 2013). SNP has a rapidly growing human population density just outside of its borders (Estes *et al.*, 2012). The phenomenon of higher population density around protected areas is not unique to SNP; in fact, this pattern is evident in most countries in Africa and South America (Wittemyer *et al.*, 2008). Though this does not indubitably lead to increased disturbance in the surrounding natural areas, when combined with greater poverty near the park, land conversion and illegal activities (such as poaching and illegal grazing) tend to concentrate around the park boundaries (Estes *et al.*, 2012). Furthermore, Veldhuis *et al.* (2019) showed that intrusions of human activities into SNP are also concentrated at its borders. These intrusions can have far-reaching effects in the Serengeti ecosystem, such as displacing wildlife and reducing soil carbon storage. Our results indicate that the concentration of human activities and disturbances around the park boundaries, coined the ‘Serengeti squeeze’, could result in elevated FGM concentrations in impala living closer to the park boundary (Veldhuis *et al.*, 2019).

Contrary to our predictions, impala in most study areas with higher protection and reduced human land use practices did not have lower FGM levels. We observed large variation in impala FGM concentrations within the national park, with nSNP having significantly lower FGM levels and impala in sSNP tending to have higher FGM levels to those in cSNP (Fig. 3C). This variation within the park could be partly due to varying levels of illegal poaching in SNP; however, recent studies are lacking to confirm this. Strikingly, impala in LGCA and GIGR, where they are arguably most affected by human disturbance, had significantly lower FGM levels than those in cSNP. Comparing GC levels in populations between management areas has given counterintuitive results before, indicating that the relationship between human activities and FGM levels in wild populations are not straightforward. African elephants living on communal lands where human activities and livestock are present did not show elevated FGM levels compared to those in protected areas (Ahlering *et al.*, 2013). Similarly, forest elephants (*Loxodonta cyclotis*) were found to have lower FGM concentrations outside of protected areas (Munshi-South *et al.*, 2008). Indeed, below, we discuss two mechanisms by which human activities could lower FGM levels in impala.

Using coarse-scale artificial spatial categorizations such as ‘inside vs outside a protected area’, however, might not fully represent the variation in FGM levels. Combining with or using instead relevant spatially explicit proxies of human disturbance, such as settlement density and proximity to protected area boundary, could perhaps provide better insight in FGM variation. Although environmental proxies such as NDVI are globally available at a high spatial and temporal resolution, this is often not the case for proxies of human disturbance. Especially for studies covering a large temporal and spatial extent such as presented here, accurate data on human disturbance is usually not available. The proxies of human disturbance presented in this study lack temporal resolution; however, they are unlikely to vary considerably within and between years; for example, impala residing in areas with high settlement density are likely to experience human disturbance throughout the year.

### Can human protection offset human disturbance?

We found that NDVI was a clear driver of FGM levels in impala, explaining 20% of the variation in FGM levels (while the full model explained 28%). Although the effect was comparatively weak, human disturbance did significantly increase FGM levels in impala. We found no evidence of an interaction between NDVI and human disturbance, however, suggesting that the effects of human disturbance might be masked by the more important stressor of low forage quality (Fig. 4). Taken together, our results indicate that impala will have higher FGM levels when lacking nutritious vegetation even when in areas without any human disturbance. In other words, impala residing in human disturbed areas *with* plenty of nutritious forage will exhibit lower FGM levels than those impala in protected areas *without* good quality forage. Pokharel *et al.* (2018) found that crop-raiding Asian elephants, which are predicted to have higher FGM levels due to their increased interaction with humans (*see*Ahlering *et al*., 2011), actually had lower FGM levels than elephants in the protected area. They found that crop-raiding elephants utilized more nutritious food sources, shown in part by higher NDVI values of the human-dominated areas. They conclude that improved diet could potentially function as a ‘pacifier’ against human-induced stress. Compared to SNP, mean NDVI in LGCA and GIGR was indeed significantly higher (Supplementary Fig. S1). These differences in NDVI could perhaps partly explain our results (see Supplementary Information S3).

Additionally, compared to SNP, surrounding areas such as LGCA also have considerably lower densities of large predators (*personal communication*). Studies have shown that GC levels can increase with higher perceived predation pressure (Clinchy *et al.*, 2013). Increased predation risk was also shown to considerably increase FGM concentrations in snowshoe hares, regardless of season and even during low predator density and low food quality (Sheriff *et al.*, 2011b). On the other hand, Chizzola *et al*. (2018) did not find a significant difference in FGM levels of impala and blue wildebeest living in areas with or without lions. Similarly, plains zebra (*Equus quagga*) living with lions did not have significantly higher FGM levels (Périquet *et al.*, 2017). Clearly, more studies are needed to disentangle the effect of predation risk on FGM (Boonstra, 2013). However, since large carnivores are abundant in the Serengeti—the park boasts one of the largest populations of lion (Swanson *et al.*, 2014)—and these predators are largely absent in human-dominated areas such as LGCA; this disparity could partly explain why impala in LGCA had lower FGM levels than those in cSNP. However, although human disturbance may influence FGM levels on an immediate level—perhaps functioning as a ‘human-shield’ by reducing predator density (Berger, 2007)—we propose that in the long term, the effect of forage quality far outweighs such disturbance for ungulates in the Serengeti ecosystem.

## Conclusion

Here we show how the interaction between proxies of environmental and anthropogenic factors affects FGM levels in a wild ungulate. Our results demonstrate the importance of forage quality in determining FGM levels in impala, much more so than human disturbance. The proxies of human disturbance used in this study, however, did elicit higher FGM levels in impala. Climate change is predicted to increase the frequency of extreme weather events, potentially leading greater seasonal fluctuations forage quality. Though certain human activities undoubtedly have negative consequences for wildlife populations in protected areas such as in the Serengeti ecosystem, our results suggest that management should focus on ensuring forage quality through drought mitigation, habitat protection and sustainable land use, if they are to protect and conserve wild ungulates populations.

## Funding

This work was supported by the European Union’s Horizon 2020 research and innovation program [Grant No. 641918 (AfricanBioServices)] and through a travel grant by the Department of Biology at the Norwegian University of Science and Technology (NTNU) [Grant No. N11005].

## Supplementary Material

revised_supplementary_information_coz117
