# Supplementary material for: Consequences of climate-induced vegetation changes exceed those of human disturbance for wild impala in the Serengeti ecosystem
Source: Conserv Physiol. 2020 Jan 21;8(1):coz117. doi: 10.1093/conphys/coz117 (PMC7246078; doi:10.1093/conphys/coz117)
Supplement: revised_supplementary_information_coz117 [file revised_supplementary_information_coz117.docx]

# Supplementary Information

**Table S1. Number of samples collected in each collection period (CP) and in each study area. Numbers in brackets are those collected in the rainy season.**

| CP \ Area | cSNP | wSNP | nSNP | sSNP | GIGR | IWMA | LGCA | *total* |
| --- | --- | --- | --- | --- | --- | --- | --- | --- |
| 2012 | 80 | 34 | 26 | 17 | 18 | 1 | 15 | 191 |
| 2016 | 45(6) | 8 | 6 | 0 | 18 | 14 | 51(12) | 142(18) |
| 2017 | 90(54) | 22 | 0 | 0 | 12 | 6 | 16 | 214(54) |
| 2018 | 38(12) | 17 | 13 | 15 | 4 | 19 | 54(50) | 160(62) |
| *total* | 253(72) | 81 | 45 | 32 | 52 | 40 | 136(62) | 639(134) |

**S1. Calculation of percent woody cover and adjusting NDVI estimates**

The data on percent woody cover (WC) were retrieved from the online Application for Extracting and Exploring Analysis Ready Samples (AppEEARS), courtesy of the NASA (<https://lpdaacsvc.cr.usgs.gov/appeears/>). These data are annual estimates of percent woody cover of the study are (Dimiceli et al., 2015). We extracted WC estimates of sample locations from the data specific to the year of collection (i.e. 2012, 2016, 2017, or 2018). WC estimates at sample locations were low (mean ± SD = 5.6% ± 3.2; N = 693). Even so, we created a separate variable with adjusted NDVI values by reducing the original NDVI estimate by a percentage equal to the WC. For example, if a datapoint had an NDVI = 0.2 and a WC = 10%, the adjusted NDVI = 0.18. Comparing the two full models including either NDVI or adjusted NDVI revealed no significant improvement of the model fit (AICc = 1340.191 and 1340.667, resp.).

**S2. Additional information on potential confounders**

Due to the fact that GCs are metabolized in the gut, factors affecting their metabolism could also affect FGM concentrations without reflecting a change in plasma GCs (Goymann, 2012). For example, it is possible that due to their diet change in the dry season (Wronski, 2002), impala ingest more fiber which could slow digestion, thus increasing GC metabolism and hence affecting FGM concentrations (Dantzer et al., 2011). In addition, impala tend to ingest more forage in the dry season than in the rainy season (Jarman & Jarman, 1973), potentially lowering FGM levels in the feces. However, these findings are ambiguous at most and are unlikely to affect our interpretation of the results as these effects, if present, would affect impala in all study areas similarly. The sample was collected for analysis if it had not been urinated on and less than 1h had passed before we were able to retrieve it (if for example we had to wait for the group to move). Fecal pellets from different parts of the pile were collected to account for any potential variation in hormone metabolite concentrations.

Studies show that in order to correctly interpret variations in FGM concentrations, it is important to either account for potential confounders in the study design, or by including such factors as predictors explaining FGM concentrations. Here, we included time-of-day as fixed predictor, since, although FGM concentrations reflect a cumulative measure of plasma GC over a certain timeframe, they have been shown to significantly change with an animal’s diurnal activity, increasing FGM in dawn and dusk (Baker, Gobush, & Vynne, 2013; Palme, 2019). Our results corroborate this as FGM levels increased significantly in early and late hours of the day.

However, certain confounders are more difficult to account for to quantify than others. By including only adult females that were not visibly pregnant, we attempted to minimize the effect of reproductive status in impala, which can significantly affect GC concentration in plasma and feces (Dantzer, Fletcher, Boonstra, & Sheriff, 2014; Edwards & Boonstra, 2018). We cannot exclude, however, the possibility that certain impala were pregnant. In fact, it is likely that many of the individuals we sampled were at varying stages of their pregnancy, since in the subtropical Serengeti region, there is no well-defined breeding season and impala give birth throughout the year (Jarman & Jarman, 1973; Schenkel, 1965). However, this variation should be similar throughout the different years and areas, so failing to properly account for reproductive status is, other than introducing error or random variation, unlikely to affect our interpretation of the results.

**S3. NDVI differences between study areas**

To test whether the unexpected result, that impala in more human disturbed areas showed lower stress levels, could be partly explained by a difference in forage availability (as per Pokharel *et al.* 2018), we modelled the variation in NDVI by land use area. We used a linear mixed effect model with NDVI as response variable, land use area and season (Wet – Dry) as fixed effects, and collection period (CP) as random effects to obtain normally distributed residuals that showed no pattern in the residual pot. The model had a marginal R^2^ of 7.8%, indicating that the combination of land use area and season did not explain much variation in NDVI. Compared to cSNP, mean NDVI in LGCA and GIGR was significantly higher; NDVI was also significantly higher in nSNP and sSNP compared to cSNP (Fig. S1).

***
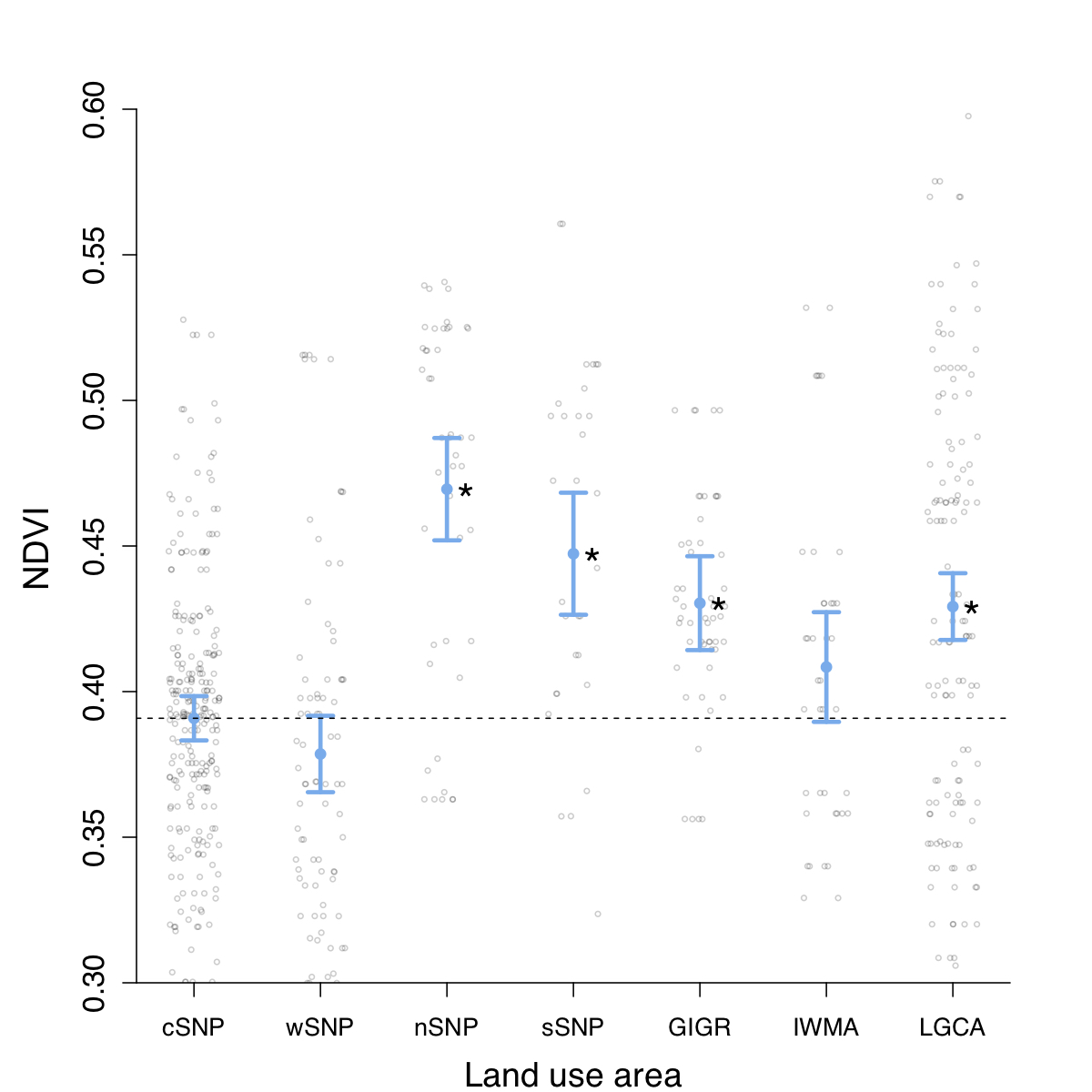
***

***Fig. S1 Mean NDVI in different land use areas****. The effect of land use area on normalized difference vegetation index (NDVI) values. Dashed line indicates reference (i.e. cSNP). Error bars show standard error of the estimate, star indicates significant difference (p < 0.001).*

**Table S2 Correlation matrix of fixed effects in final model.**

|  | | (Intercept) | NDVI | Settlement density | Land use area | | | | | | Distance to SNP (lin.) | Distance to SNP (qua.) | Time-of-day (lin.) | Time-of-day (qua.) | Rainfall Low |
| --- | --- | --- | --- | --- | --- | --- | --- | --- | --- | --- | --- | --- | --- | --- | --- |
|  |  |  |  |  | wSNP | nSNP | sSNP | GIGR | IWMA | LGCA |  |  |  |  |  |
| NDVI | | -0.709 |  |  |  |  |  |  |  |  |  |  |  |  |  |
| Settlement density | | 0.055 | 0.101 |  |  |  |  |  |  |  |  |  |  |  |  |
| Land use area | wSNP | -0.23 | 0.011 | -0.456 |  |  |  |  |  |  |  |  |  |  |  |
|  | nSNP | -0.052 | -0.24 | -0.154 | 0.345 |  |  |  |  |  |  |  |  |  |  |
|  | sSNP | 0.046 | -0.167 | -0.067 | 0.06 | 0.115 |  |  |  |  |  |  |  |  |  |
|  | GIGR | -0.13 | -0.163 | -0.227 | 0.418 | 0.426 | -0.026 |  |  |  |  |  |  |  |  |
|  | IWMA | -0.17 | -0.048 | -0.005 | 0.325 | 0.353 | -0.041 | 0.457 |  |  |  |  |  |  |  |
|  | LGCA | -0.127 | -0.257 | -0.265 | 0.419 | 0.548 | 0.038 | 0.613 | 0.495 |  |  |  |  |  |  |
| Distance to SNP (lin.) | | 0.082 | 0.012 | -0.492 | 0.001 | -0.215 | 0.266 | -0.388 | -0.445 | -0.487 |  |  |  |  |  |
| Distance to SNP (qua.) | | -0.11 | 0.134 | -0.217 | 0.32 | 0.09 | -0.357 | 0.213 | 0.208 | -0.013 | -0.003 |  |  |  |  |
| Time-of-day (lin.) | | 0.066 | -0.031 | 0.031 | -0.047 | -0.04 | -0.041 | 0.026 | -0.024 | -0.033 | -0.044 | 0.028 |  |  |  |
| Time-of-day (qua.) | | 0.036 | -0.136 | -0.075 | 0.12 | 0.048 | 0.124 | 0.162 | 0.048 | 0.168 | -0.064 | -0.04 | 0.016 |  |  |
| Rainfall | Low | -0.087 | -0.056 | 0.027 | -0.108 | 0.015 | -0.012 | -0.061 | -0.138 | 0.09 | 0.107 | -0.081 | -0.123 | -0.009 |  |
|  | High | -0.083 | -0.109 | -0.139 | 0.03 | 0.113 | 0.068 | 0.114 | 0.02 | 0.101 | 0.145 | -0.117 | -0.064 | 0.038 | 0.444 |

# References

Baker, M. R., Gobush, K. S., & Vynne, C. H. (2013). Review of factors influencing stress hormones in fish and wildlife. *Journal for Nature Conservation*, *21*(5), 309–318. doi: 10.1016/j.jnc.2013.03.003

Dantzer, B., Fletcher, Q. E., Boonstra, R., & Sheriff, M. J. (2014). Stress in Vertebrates Measures of physiological stress: a transparent or opaque window into the status, management and conservation of species? *Conservation Physiology*, *2*, 1–18. doi: 10.1093/conphys/cou023.

Dantzer, B., McAdam, A. G., Palme, R., Boutin, S., & Boonstra, R. (2011). How does diet affect fecal steroid hormone metabolite concentrations? An experimental examination in red squirrels. *General and Comparative Endocrinology*, *174*(2), 124–131. doi: 10.1016/j.ygcen.2011.08.010

Dimiceli, C., Carroll, M., Sohlberg, R., Kim, D. H., Kelly, M., & Townshend, J. R. G. (2015). *MOD44B MODIS/Terra Vegetation Continuous Fields Yearly L3 Global 250m SIN Grid V006*. doi: 10.5067/MODIS/MOD44B.006

Edwards, P. D., & Boonstra, R. (2018). Glucocorticoids and CBG during pregnancy in mammals: diversity, pattern, and function. *General and Comparative Endocrinology*, *259*, 122–130. doi: 10.1016/j.ygcen.2017.11.012

Goymann, W. (2012). On the use of non-invasive hormone research in uncontrolled, natural environments: The problem with sex, diet, metabolic rate and the individual. *Methods in Ecology and Evolution*, *3*(4), 757–765. doi: 10.1111/j.2041-210X.2012.00203.x

Jarman, M. V., & Jarman, P. J. (1973). Daily activity of impala. *African Journal of Ecology*, *11*(1), 75–92. doi: 10.1111/j.1365-2028.1973.tb00074.x

Palme, R. (2019). Non-invasive measurement of glucocorticoids: Advances and problems. *Physiology & Behavior*, *199*, 229–243. doi: 10.1016/j.physbeh.2018.11.021

Pokharel, S. S., Singh, B., Seshagiri, P. B., & Sukumar, R. (2018). Lower levels of glucocorticoids in crop-raiders: diet quality as a potential ‘pacifier’ against stress in free-ranging Asian elephants in a human-production habitat. *Animal Conservation*, *2010*, 1–12. doi: 10.1111/acv.12450

Schenkel, R. (1965). *On Sociology and Behavour in Impala*.

Wronski, T. (2002). Feeding ecology and foraging behaviour of impala *Aepyceros melampus*. *African Journal of Ecology*, *40*(3), 205–211. doi: 10.1046/j.1365-2028.2002.00348.x
